# The evaluation of the training, “the approach to epilepsy and epileptic seizure,” which was given to teachers in Türkiye

**DOI:** 10.1002/brb3.3538

**Published:** 2024-05-23

**Authors:** Mert Kartal, Neşe Karakaş, Pınar Tuz, Gürkan Kapıkıran

**Affiliations:** ^1^ Faculty of Health Sciences, Public Health Malatya Turgut Özal University Battalgazi Malatya Türkiye; ^2^ Faculty of Medical Sciences, Public Health Malatya Turgut Özal University Battalgazi Malatya Türkiye; ^3^ Ministry of Education Huzurevleri Primary School Diyarbakır Türkiye; ^4^ Faculty of Health Sciences, Surgical Diseases Nursing Malatya Turgut Özal University Battalgazi Malatya Türkiye

**Keywords:** epilepsy, epileptic seizure, teacher, training

## Abstract

**Introduction:**

Epilepsy is the most common neurological disorder among humans after headaches. According to the World Health Organization, approximately 50–65 million individuals were diagnosed with epilepsy throughout the world, and around two million new cases of epilepsy are added to this figure every year.

**Methods:**

Designed as descriptive and cross‐sectional research, this study was performed on 132 elementary school teachers. Training on epilepsy and epileptic seizure was given to teachers. The pretest and posttest research data were collected with the face‐to‐face interview method. In this process, the epilepsy knowledge scale was used as well as a survey form that had questions designed to find out about teachers’ personal characteristics. The Statistical Package for Social Science 25.0 was utilized in the statistical analysis of research data. In the research, the statistical significance was identified if the *p*‐value was below.05 (*p* < .05).

**Results:**

Of all teachers participating in the study, 59.1% were female, 90.2% were married, and 47.7% witnessed an epilepsy seizure before. The mean of teachers’ pretest epilepsy knowledge scores was 8.43 ± 4.31 points before the training while the mean of their posttest epilepsy knowledge scores was 12.65 ± 2.48 points after the training. The difference between the means of pretest and posttest scores was statistically significant (*p* = .000). After the training, there was a statistically significant increase in means of scores obtained by teachers from each item of the epilepsy knowledge scale (*p* < .05).

**Conclusions:**

As there was a statistically significant improvement in levels of teachers’ knowledge about both epilepsy and epileptic seizure after the training, it is recommended that the training about the approach to epilepsy and epileptic seizure be given to all teachers, and additionally, including these topics in the course curricula of universities is recommended.

## Graphical abstract

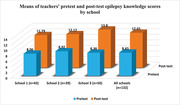

## 1 INTRODUCTION

Epilepsy is a prevalent neurological disorder, second only to headaches. The World Health Organization reports that approximately 50–65 million people worldwide have been diagnosed with epilepsy, with an additional two million new cases each year (Karakaş et al., 2022; World Health Organization, 2019). Epilepsy, which was known since the early ages of history, was perceived as dangerous and frightening by society (Yıldırım et al., 2018). Upon the review of the prevalence rate of the disease, it was discerned that the prevalence rate was 6 per thousand in developed countries and 18.5 per thousand in developing countries. In Türkiye, the prevalence rate for epilepsy was reported as 6.1–10.2 per thousand (Aksun & Yiğit, 2021; Aktürk et al., 2020). It is estimated that, among children aged below 15 years throughout the world, there were 10.5 million epilepsy patients irrespective of gender differences (Howell et al., 2016; Scheffer et al., 2017). Epilepsy is characterised by seizures that occur without warning signs. Seizures can also manifest as convulsions leading to loss of consciousness. Identifying epileptic seizures is crucial for patient quality of life and recovery (Hussein et al., 2018). In epilepsy, seizure management is one of the basic goals of the treatment. Taking the child, who has an epileptic seizure, to a safe area is the most accurate act to ensure that the child will have the seizure with a minimum amount of loss. On the other hand, wrong practices performed during the epileptic seizure lead to aspiration, fractures and dislocations, muscle contractions, and traumas in the child and, accordingly, inflict harm on the child (Wirrell, 2006). Children spend most of their time at school. For this reason, teachers in the school have great responsibilities. At the same time, teachers are likely to encounter a student who has an epileptic seizure. Proper first aid will have a positive impact on the individual's health. Students and teachers sometimes witness the sudden loss of control in a child having an epileptic seizure and are intimidated by the situation. Such situations can lead to the child's social exclusion (Savarese et al., 2015). It is possible for primary school teachers to play an important role by sharing information about epilepsy in order to solve the problem of social discrimination of children diagnosed with epilepsy in school (Assadeck et al., 2020). Therefore, it is essential for teachers who spend a significant amount of time with students at school to have knowledge about epileptic seizures. Insufficient knowledge about the appropriate practices during an epileptic seizure can cause panic among teachers. Previous research studies have shown that parents, teachers, and children diagnosed with epilepsy lack adequate knowledge about the condition, leading to difficulties (Owolabi et al., 2014; Savarese et al., 2015).

The aim of this research is to assess the effectiveness of training provided to teachers on the approach to epilepsy and epileptic seizures.

## 2 MATERIAL AND METHODS

### 2.1 Research design and sample

This research study was conducted as a descriptive and single‐group intervention. It was carried out between April and June 2022 with teachers working in the three primary schools with the highest number of students in Kayapınar, the central district of Diyarbakır province located in southeastern Türkiye. The study population consisted of 165 teachers working in these three schools. The sample size for the study was calculated using the sampling formula [*n* = *N*. *t* ^2^ *p*.*q* / (*d* ^2^.(*N* – 1) + *t* ^2^ *p*.*q*)], with α = .05, *p* = *q* = .5, and *d* = ± 0.05. The minimum number of teachers required for the study was determined to be 116. The study sample comprised 132 teachers who agreed to participate and met the inclusion criteria. The inclusion criteria were having received basic first aid training and volunteering to participate in the study. Participants who did not receive basic first aid training or did not volunteer to participate in the study were excluded. This exclusion criterion was applied to ensure the objectivity and reliability of the study results.

### 2.2 Data collection tools

The researchers used a data collection tool consisting of an 8‐question personal information form and a 16‐item epilepsy knowledge scale (EKS).

#### 2.2.1 Personal information form

The study included eight questions that aimed to determine the personal characteristics of the teachers. These questions covered gender, marital status, education branch, education level, prior experience with epilepsy (witnessing a seizure, hearing about epilepsy, researching epilepsy, or knowing someone diagnosed with epilepsy).

#### 2.2.2 The epilepsy knowledge scale (EKS)

Aydemir developed the epilepsy knowledge scale for the Turkish population, which has a triple‐Likert structure. The scale questions comprise 16 items covering causes of epilepsy, seizure triggers, social limitations resulting from epilepsy, treatment methods, and appropriate seizure interventions. The scale questions can be answered with “true,” “no idea,” and “false.” The minimum score is “0” and the maximum score is “16.” The level of knowledge about epilepsy increases with a higher score on the scale. The validity and reliability of the scale is confirmed with a Cronbach's alpha value of.72 (Aydemir, 2008). In the study, the value was recorded as 0.79.

### 2.3 Epilepsy education intervention

Epilepsy training material was prepared in line with the literature. The educational material included these questions. The content of the education material: “Prevalence of epilepsy in the world and in Türkiye,” “where epilepsy originates from,” “in whom the disease is more common,” “causes of epilepsy seizures and how they occur,” “what are the symptoms and findings that can be seen in seizure types,” “conditions that trigger seizures,” “seizure control can reduce the effects of epilepsy,” “how to apply first aid to a child with epilepsy,” “how to educate children with epilepsy if there are children with epilepsy,” “what are the sports activities that children with epilepsy can and absolutely cannot do,” and “what to do after a seizure.”

### 2.4 Data collection

The research data were collected through face‐to‐face interviews. The study's primary school teachers received detailed explanations about the research's purpose, objectives, benefits, and interview time. Informed consent was obtained from primary school teachers who wished to participate in the study after receiving all necessary information about the research. Questionnaires were distributed to primary school teachers who met the criteria for participation in the study. The pretest application included a personal information form and an epilepsy knowledge scale, which were administered under the supervision of the researchers. Following completion of the initial questionnaire, the researchers provided training on epilepsy based on relevant literature. Subsequently, primary school teachers were given the opportunity to ask questions, which were answered in a question‐answer format. The training and posttraining session lasted approximately 25 min. Following the training, the written materials were shared with primary school teachers. Those who provided their telephone numbers on the questionnaire forms were contacted 4 weeks after the pretest application to schedule an appointment. Finally, the epilepsy knowledge scale was administered again as a posttest and lasted approximately 5 min.

### 2.5 Statistical analysis

The data was evaluated using the Statistical Package for the Social Sciences IBM 25.0 (Armonk, NY: IBM Corp.) analysis programme. Statistical analyses were conducted at a 95% confidence interval and a significance level of *p* < .05. The normal distribution of the data was determined using the Kolmogorov–Smirnov test. Parametric tests were used to analyze normally distributed data. Demographic data was evaluated using percentage, mean, and standard deviation tests. Paired‐samples *t*‐test and independent‐samples *t*‐test, as well as one‐way analysis of variance (ANOVA), were used to compare nominal data. The reliability and validity of the scales used in the study were determined using Cronbach's alpha coefficient.

## 3 RESULT

**FIGURE 1:**
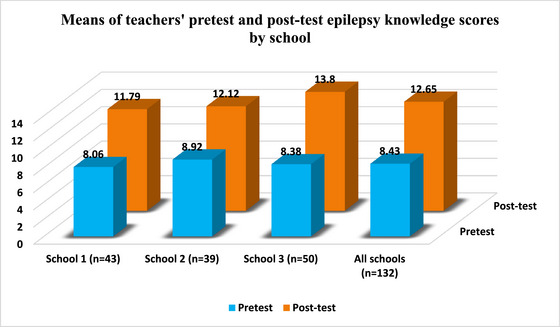
The breakdown of teachers’ mean pretest and posttest epilepsy knowledge scores by school.

Table 1 displayed the breakdown of teachers’ mean pretest and posttest epilepsy knowledge scores as per their personal characteristics. Of all participant teachers, 59.1% were female, 90.2% were married, 84.1% were core teachers, and 88.6% held bachelor's degrees. Besides, of all participant teachers, 52.3% never witnessed an epileptic seizure before, 18.9% never heard about epilepsy before, 34.1% had researched epilepsy, and 40.9% knew a person diagnosed with epilepsy. Means of all participant teachers’ pretest and posttest epilepsy knowledge scores were respectively 8.43 and 12.65 points. Upon the review of teachers’ mean pretest epilepsy knowledge scores, it is discerned that means of pretest epilepsy knowledge scores obtained by female teachers, married teachers, field teachers, and teachers with master's degrees were successively 9.02, 8.47, 9.00, and 8.93 points. Next, in the context of the examination of teachers’ mean posttest epilepsy knowledge scores, it is identified that means of posttest epilepsy knowledge scores obtained by all teachers, male teachers, married teachers, core teachers, and teachers with master's degrees were consecutively 12.65, 12.81, 12.66, 12.71, and 13.13 points. Figure 1 exhibited the breakdown of teachers’ mean pretest and posttest epilepsy knowledge scores by school. It is found that, at all three schools, there was an increase in means of teachers’ epilepsy knowledge scores after the training.

**TABLE 1:** The breakdown of teachers’ mean pretest and posttest epilepsy knowledge scores as per their personal characteristics.

Personal characteristics	*N*	%	Pretest	Posttest
			(®𝒙 **± SD)**	(®𝒙 **± SD)**
**Gender**				
Female	78	59.1	9.02 ± 4.15	12.53 ± 2.58
Male	54	40.9	7.59 ± 4.43	12.81 ± 2.35
			*t*: 1.896, *p*:.060	*t*: 0.626, *p*:.532
**Marital status**				
Married	119	90.2	8.47 ± 4.27	12.66 ± 2.46
Single	13	9.8	8.15 ± 4.79	12.53 ± 2.75
			*t*: –0.251, *p*:.803	*t*: –0.172, *p*:.864
**Education level**				
Bachelor's degree	117	88.6	8.37 ± 4.24	12.58 ± 2.51
Master's degree	15	11.4	8.93 ± 4.96	13.13 ± 2.23
			*t*: –0.470, *p*:.639	*t*: –0.796, *p*:.427
**Branch**				
Core teacher	111	84.1	8.33 ± 4.30	12.71 ± 2.36
Field teacher	21	15.9	9.00 ± 4.41	12.33 ± 3.10
			*t*: –0.648, *p*:.518	*t*: 0.638, *p*:.525
**Status of witnessing an epilepsy seizure before**				
Yes	63	47.7	9.31 ± 4.21	12.77 ± 2.28
No	69	52.3	7.63 ± 4.27	12.53 ± 2.66
			*t*: 2.270, * ***p*:.025**	*t*: 0.556, *p*:.579
**Status of hearing about epilepsy before**				
Yes	107	81.1	8.93 ± 4.29	12.38 ± 2.56
No	25	18.9	6.32 ± 3.76	13.80 ± 1.70
			*t*: 2.799, * ***p*:.006**	*t*: −2.622, * ***p*:.010**
**Status of researching epilepsy**				
Yes	45	34.1	10.31 ± 4.32	12.44 ± 2.49
No	87	65.9	7.47 ± 3.99	12.75 ± 2.49
			*t*: 3.762, * ***p*:.000**	*t*: –0.687, *p*:.493
**Status of knowing a person diagnosed with epilepsy**				
Yes	54	40.9	9.97 ± 4.16	12.24 ± 2.71
No	78	59.1	7.79 ± 4.31	12.93 ± 2.29
			*t*: 2.090, * ***p*:.039**	*t*: −1.588, *p*:.115

*Note*: Mean is represented by X̄ and standard deviation is represented by SD.

*t*: independent sample *t*‐test.

*p < .05.

The bold value is statistically significant results.

Table 2 showed the comparison of means of teachers’ pretest and posttest epilepsy knowledge scores, and it was discerned that the mean of teachers’ posttest scores was higher than the mean of their pretest scores and this difference between the means of pretest and posttest scores was statistically significant (*p* = .000).

**TABLE 2:** The comparison of means of teachers’ pretest and posttest epilepsy knowledge scores.

Epilepsy knowledge	Pretest scores	Posttest scores
	$\bar{x}$ **+ SD/Min–Max**	$\bar{x}$ **+ SD/Min–Max**
Epilepsy knowledge scores	8.43 ± 4.31 (0–16)	12.65 ± 2.48 (0–16)
	*t* = −9.376, ***p* = .000**	

The bold value is statistically significant results.

Table 3 indicated the means of pretest and posttest epilepsy knowledge scores obtained by teachers from each EKS item. In the posttest phase after the training, there was a statistically significant increase in the means of scores obtained by teachers from each EKS item (*p* < .05).

**TABLE 3:** The breakdown of means of pretest and posttest epilepsy knowledge scores obtained by teachers from each EKS item.

EKS items		The mean of scores		*p*
		Pretest	Posttest	
1	Epilepsy has many different types.	0.36	0.77	**.000**
2	Most people with epilepsy can work.	0.64	0.93	**.000**
3	Most children with epilepsy can go to public school.	0.66	0.95	**.000**
4	Patients with epilepsy can be dangerous to others during a seizure.	0.65	0.78	**.024**
5	Some seizures may last for a matter of seconds.	0.37	0.65	**.000**
6	For most patients with epilepsy, seizures can be controlled with a drug.	0.58	0.84	**.000**
7	Brain surgery can be used to treat epilepsy in some cases.	0.18	0.48	**.000**
8	Most people with epilepsy have normal intelligence.	0.73	0.96	**.000**
9	Patients with epilepsy can be as successful at work as others.	0.71	0.93	**.000**
10	An epileptic seizure is caused by an abnormal function of the nerve cells in the brain.	0.44	0.84	**.000**
11	Epilepsy is a kind of incurable disorder.	0.53	0.74	**.001**
12	Inadequate sleep, stress, and taking alcohol can cause a seizure.	0.62	0.78	**.008**
13	When you see a person having a seizure, you can stop the seizure by giving him/her an onion to smell.	0.37	0.80	**.000**
14	Patients with epilepsy can lead normal lives.	0.72	0.96	**.000**
15	Some kinds of seizures can be hardly noticed by others.	0.39	0.78	**.000**
16	When you see a person having a seizure, you should spill water on his/her face to stop the seizure.	0.40	0.80	**.000**

The bold value is statistically significant results.

## 4 DISCUSSION

In this study where we aimed to evaluate elementary school teachers’ epilepsy knowledge levels before and after giving an epilepsy training, it was identified that teachers had insufficient levels of knowledge about epilepsy before the training. In the relevant literature, it was emphasized that the level of general epilepsy knowledge was low (Al‐Hashemi et al., 2016; Buccheri & Quattropani, 2015; Kampra et al., 2016; Khanal et al., 2015; Savarese et al., 2015). Also, in a study performed with 135 teachers, it was found that 41% of the teachers had knowledge about epilepsy (Berhe et al., 2017). Moreover, in the study conducted by Yeşilyurt on guidance counselors’ epilepsy knowledge levels, it was discerned that only 17.8% of the guidance counselors had accurate knowledge about epilepsy (Yeşilyurt, 2019). Besides, in another study performed on primary school teachers, it was found that 58% of the teachers did not accurately know what to do during an epilepsy crisis (Abulhamail et al., 2014). It is considered that the level of knowledge about this topic was low in our research as 65.9% of the participant teachers had not researched epilepsy and a significant part of them said that they never heard about epilepsy before.

Next, the mean of epilepsy knowledge scores of teachers who participated in our study was low in the pretest phase before the training (8.43 points) whereas the mean of their epilepsy knowledge scores increased in the posttest phase after the training (12.65 points) and this difference in means of teachers’ pretest and posttest scores was statistically significant. Additionally, upon the examination of means of scores per EKS item, it was discerned that means of epilepsy knowledge scores obtained by teachers from each EKS item increased in the posttest phase after the training and these differences in means of teachers’ pretest and posttest scores were statistically significant. In the study by Yeşilyurt, it was stated that teachers responded by saying, “I try to open the child's chin” and “I make the child smell an onion” regarding the approach adopted toward a child having an epilepsy seizure, and it was underlined that teachers needed training on this topic (Yeşilyurt, 2019). In parallel to these responses given in the study by Yeşilyurt, the number of teachers who responded that they would stop the epileptic seizure by making the child smell an onion was high in our study before the training, on the other hand, after the training, there was a statistically significant decrease in the number of teachers giving this response (the mean of pretest scores = 0.37, the mean of posttest scores = 0.80). Likewise, in our study, there were statistically significant improvements also in responses given to the EKS item, “When you see a person having a seizure, you should spill water on his/her face to stop the seizure” and other EKS items. These results highlight the necessity and effectiveness of the training. Moreover, 47.7% of the teachers participating in our study stated that they witnessed an epileptic seizure before. Upon the review of the relevant literature, we find that the results in the literature are similar to this finding of our study. In this regard, 62.2% of the teachers in the study by Üçer et al., 62.9% of the teachers in the study by Yaman et al., and 62.8% of the teachers in the study by Mecarelli et al. reported witnessing an epilepsy seizure before (Mecarelli et al., 2011; Üçer et al., 2016; Yaman et al.). In numerous studies that analyzed teachers’ epilepsy‐related knowledge, attitudes, and practices, it was advocated that teachers did not have sufficient knowledge about epilepsy and needed to receive training about it (Alamri et al., 2018; Al‐Harbi et al., 2018; Alkhotani et al., 2019; Alqahtani, 2015; Kampra et al., 2016). Having accurate knowledge about what to do during epileptic seizures will enable teachers to assure that the seizure will happen with no harm to the child and without panic and families with children diagnosed with epilepsy will not feel anxiety.

Low‐risk sports such as team sports, dance, and golf are recommended for students with epilepsy. However, an individual risk assessment should always be carried out for medium‐risk sports such as skiing, gymnastics and swimming, and high‐risk sports such as climbing, motor sports and surfing (Howard et al., 2004; van den Bogard et al., 2020). Teachers' knowledge of the condition is important for the correct guidance of students in sports and exercise.

The research was limited to teachers working in three primary schools in a province in Türkiye. Middle schools and high schools were not included in this study. Therefore, it is not possible to generalize for all teachers in Türkiye. It is a rare study showing that teachers’ knowledge about epilepsy and epileptic seizures is not sufficient. This is a study that reveals the necessity of providing training to teachers about this disease, as there is a high probability of encountering students who are sick or have seizures. It is a study that reveals the importance of education with pretest and posttest.

## 5 CONCLUSION

At the end of this research where we targeted to evaluate the effectiveness of the training given to elementary school teachers, it was identified that teachers did not have a sufficient level of knowledge about epilepsy before the training. Almost half of the teachers who participated in the study reported encountering a student with epileptic seizures at least once. The test results applied to the same teachers before the training revealed insufficient knowledge about epilepsy and how to approach seizures. On the other hand, after the epilepsy training offered to teachers, it was discerned that there was a statistically significant improvement in levels of teachers’ knowledge about epilepsy and epileptic seizure. In this direction, It is recommended that a course entitled “Approach to Students with Chronic Diseases” be added to the university curriculum. Additionally, all teachers should receive training on epilepsy and how to approach students with epilepsy under the sub‐heading “Epilepsy Education and Approaches to Students with Epilepsy” as part of this course. The proposed improvements aim to increase teachers’ self‐confidence, ensure the social and psychological empowerment of students who feel safe, minimize harm to oneself and the environment with effective first aid during seizures, and decrease society's anxiety about sick children while they are at school.

## AUTHOR CONTRIBUTIONS

**Mert Kartal**: Conceptualization; methodology; investigation; validation; software; formal analysis; funding acquisition; project administration; writing—review and editing; writing—original draft; supervision. **Neşe Karakaş**: Conceptualization; methodology; investigation; writing—original draft; visualization. **Pınar Tuz**: Data curation; conceptualization; methodology; software; investigation; funding acquisition; writing—original draft. **Gürkan Kapıkıran**: Writing—review and editing; formal analysis; conceptualization; investigation; writing—original draft; supervision; project administration; methodology.

## CONFLICT OF INTEREST STATEMENT

None of the authors has any conflict of interest to disclose.

## FUNDING

This research received no specific grant from any funding agency in the public, commercial, or not‐for‐profit sectors.

### PEER REVIEW

The peer review history for this article is available at https://publons.com/publon/10.1002/brb3.3538.

## Data Availability

The datasets analyzed during the current study are available from the corresponding authors on reasonable request

## References

[brb33538-bib-0001] Abulhamail, A. S. , Al‐Sulami, F. E. , Alnouri, M. A. , Mahrous, N. M. , Joharji, D. G. , Albogami, M. M. , & Jan, M. M. (2014). Primary school teacher's knowledge and attitudes toward children with epilepsy. Seizure: The Journal of the British Epilepsy Association, 23(4), 280–283. 10.1016/j.seizure.2013.12.010 24445017

[brb33538-bib-0002] Aksun, Z. Ö. , & Yiğit, A. (2021). Investigation of behaviors of the epilepsy patients’ relatives during seizure and its association with their knowledge regarding epilepsy. Stroke; A Journal of Cerebral Circulation, 3, 13.

[brb33538-bib-0003] Aktürk, T. , Tanık, N. , Saçmacı, H. , Chia, Z.‐J. , & Lim, K.‐S. (2020). Validity and reliability of the Turkish version of Public Attitudes Toward Epilepsy scale. Epilepsy & Behavior, 111, 107245. 32693372 10.1016/j.yebeh.2020.107245

[brb33538-bib-0004] Alamri, S. , Alghamdi, A. , & Al Quait, A. (2018). What Saudi teachers know about epilepsy: A cross‐sectional study of Tabuk City. Epilepsy & Behavior, 89, 169–172. 30419429 10.1016/j.yebeh.2018.10.024

[brb33538-bib-0005] Al‐Harbi, A. , Alsaid, L. , & Parameaswari, P. (2018). Primary school female teachers’ knowledge, attitude, and practice toward students with epilepsy in Riyadh, Saudi Arabia. Journal of Family Medicine and Primary Care, 7(2), 331. 10.4103/jfmpc.jfmpc_58_18 PMC6060931 30090773

[brb33538-bib-0006] Al‐Hashemi, E. , Ashkanani, A. , Al‐Qattan, H. , Mahmoud, A. , Al‐Kabbani, M. , Al‐Juhaidli, A. , Jaafar, A. , & Al‐Hashemi, Z. (2016). Knowledge about epilepsy and attitudes toward students with epilepsy among middle and high school teachers in Kuwait. International journal of Pediatrics, 2016, 1–15. 10.1155/2016/5138952 PMC4925992 27403170

[brb33538-bib-0007] Alkhotani, A. M. , Almalki, W. M. , Alkhotani, A. M. , & Turkistani, M. A. (2019). Makkah female teachers’ knowledge of seizure first aid. Epilepsy & Behavior, 98, 10–13. 31299526 10.1016/j.yebeh.2019.05.047

[brb33538-bib-0008] Alqahtani, J. M. (2015). Knowledge and practice of schoolteachers towards students with epilepsy in Khamis Mushate, Southern Saudi Arabia. Journal of Family & Community Medicine, 22(3), 163. 26392797 10.4103/2230-8229.163034 PMC4558738

[brb33538-bib-0009] Assadeck, H. , Toudou Daouda, M. , Moussa Konate, M. , Mamadou, Z. , Douma Maiga, D. , & Sanoussi, S. (2020). Knowledge, attitudes, and practices with respect to epilepsy among primary and secondary school teachers in the city of Niamey, Niger. Brain and Behavior, 10(3), e01539. 10.1002/brb3.1539 31989794 PMC7066328

[brb33538-bib-0010] Aydemir, N. (2008). Developing two different measures for assessing knowledge of and attitudes toward epilepsy for the Turkish population. Epilepsy & Behavior, 12(1), 84–89. 17974487 10.1016/j.yebeh.2007.07.018

[brb33538-bib-0011] Berhe, T. , Yihun, B. , Abebe, E. , & Abera, H. (2017). Knowledge, attitude, and practice about epilepsy among teachers at Ethio‐National School, Addis Ababa, Ethiopia. Epilepsy & Behavior, 70, 150–153. 28427024 10.1016/j.yebeh.2017.02.009

[brb33538-bib-0012] Buccheri, T. , & Quattropani, M. C. (2015). Perception of, attitudes toward, and knowledge of epilepsy among teachers and high school and college students in Sicily. Epilepsy & Behavior, 53, 43–50. 26519665 10.1016/j.yebeh.2015.09.033

[brb33538-bib-0013] Howard, G. M. , Radloff, M. , & Sevier, T. L. (2004). Epilepsy and sports participation. Current Sports Medicine Reports, 3(1), 15–19. 10.1249/00149619-200402000-00004 14728909

[brb33538-bib-0014] Howell, K. B. , Harvey, A. S. , & Archer, J. S. (2016). Epileptic encephalopathy: Use and misuse of a clinically and conceptually important concept. Epilepsia, 57(3), 343–347. 10.1111/epi.13306 26778176

[brb33538-bib-0015] Hussein, R. , Palangi, H. , Ward, R. , & Wang, Z. J. (2018). Epileptic seizure detection: A deep learning approach. *arXiv preprint arXiv:1803.09848*.

[brb33538-bib-0016] Kampra, M. , Tzerakis, N. G. , Losidis, S. , Katsarou, E. , Voudris, K. , Mastroyianni, S. , Mouskou, S. , Siatouni, A. , & Gatzonis, S. (2016). Teachers' knowledge about epilepsy in Greece: Information sources and attitudes towards children with epilepsy during school time. Epilepsy & Behavior, 60, 218–224. 27240308 10.1016/j.yebeh.2016.04.004

[brb33538-bib-0017] Karakaş, N. , Sarıtaş, S. Ç. , Aktura, S. Ç. , Karabulutlu, E. Y. , & Oruç, F. G. (2022). Investigation of factors associated with stigma and social support in patients with epilepsy in Turkey: A cross‐sectional study. Epilepsy & Behavior, 128, 108572. 35123241 10.1016/j.yebeh.2022.108572

[brb33538-bib-0018] Khanal, K. , Maharjan, R. , Pokharel, B. R. , & Sanjel, S. (2015). School teachers’ knowledge about epilepsy in Kathmandu Metropolitan City. Kathmandu University Medical Journal, 13(4), 316–322. 10.3126/kumj.v13i4.16830 27423281

[brb33538-bib-0019] Mecarelli, O. , Capovilla, G. , Romeo, A. , Rubboli, G. , Tinuper, P. , & Beghi, E. (2011). Knowledge and attitudes toward epilepsy among primary and secondary schoolteachers in Italy. Epilepsy & Behavior, 22(2), 285–292. 21795121 10.1016/j.yebeh.2011.06.019

[brb33538-bib-0020] World Health Organization . (2019). Epilepsy: A public health imperative. World Health Organization.

[brb33538-bib-0021] Owolabi, L. F. , Shehu, N. M. , & Owolabi, S. D. (2014). Epilepsy and education in developing countries: A survey of school teachers’ knowledge about epilepsy and their attitude towards students with epilepsy in Northwestern Nigeria. The Pan African Medical Journal, 18, 255. 10.11604/pamj.2014.18.255.3607 25489360 PMC4258207

[brb33538-bib-0022] Savarese, G. , Carpinelli, L. , D'elia, D. , & Coppola, G. (2015). Teachers of various school grades and representations of epilepsy: Problems, relational aspects and perspectives of life quality. Italian Journal of Pediatrics, 41(1), 1–5. 10.1186/s13052-015-0177-8 26437951 PMC4595060

[brb33538-bib-0023] Scheffer, I. E. , Berkovic, S. , Capovilla, G. , Connolly, M. B. , French, J. , Guilhoto, L. , Hirsch, E. , Jain, S. , Mathern, G. W. , Moshé, S. L. , Nordli, D. R. , Perucca, E. , Tomson, T. , Wiebe, S. , Zhang, Y.‐H. , & Zuberi, S. M. (2017). ILAE classification of the epilepsies: Position paper of the ILAE Commission for Classification and Terminology. Epilepsia, 58(4), 512–521. 10.1111/epi.13709 28276062 PMC5386840

[brb33538-bib-0024] Üçer, H. , Sucakli, M. H. , Celik, M. , & Keten, H. S. (2016). Primary school teachers' knowledge, attitudes and behaviors about childhood epilepsy. CUKUROVA MEDICAL JOURNAL, 41(3), 491–497.

[brb33538-bib-0025] van den Bogard, F. , Hamer, H. M. , Sassen, R. , & Reinsberger, C. (2020). Sport and physical activity in epilepsy. Deutsches Arzteblatt international, 117(1‐2), 1–6. 32008605 10.3238/arztebl.2020.0001 PMC7008149

[brb33538-bib-0026] Wirrell, E. C. (2006). Epilepsy‐related injuries. Epilepsia, 47, 79–86. 10.1111/j.1528-1167.2006.00666.x 17044832

[brb33538-bib-0027] Yaman, S. , Arıkan, D. , Çelebioğlu, A. , Özyazıcıoğlu, N. , & Güdücü, F. (2006). Teachers' knowledge and attitude regard to epilepsy. Journal of Nursology, 4(1), 18–24.

[brb33538-bib-0028] Yeşilyurt, F. (2019). Investigating the knowledge and behavior of guidance counselors regarding epilepsy. Archives of Epilepsy, 25, 141–146.

[brb33538-bib-0029] Yıldırım, Z. , Ertem, D. H. , Dirican, A. C. , & Baybaş, S. (2018). Stigma accounts for depression in patients with epilepsy. Epilepsy & Behavior, 78, 1–6. 29161628 10.1016/j.yebeh.2017.10.030

